# Proteomic Analysis Reveals Coordinated Regulation of Anthocyanin Biosynthesis through Signal Transduction and Sugar Metabolism in Black Rice Leaf

**DOI:** 10.3390/ijms18122722

**Published:** 2017-12-15

**Authors:** Linghua Chen, Yining Huang, Ming Xu, Zuxin Cheng, Jingui Zheng

**Affiliations:** 1 College of Life Sciences, Fujian Agriculture and Forestry University, Fuzhou 350002, China; clinghua_91@126.com; 2 Jinshan College of Fujian Agriculture and Forestry University, Fuzhou 350002, China; 3 Department of Food and Biology Engineering, Zhangzhou Institute of Technology, Zhangzhou 363000, China; huangyining8@126.com; 4 College of Crop Science, Fujian Agriculture and Forestry University, Fuzhou 350002, China; xmfau@163.com (M.X.); chengzuxin@163.com (Z.C.)

**Keywords:** proteomic, anthocyanin, leaf, black rice, iTRAQ

## Abstract

Black rice (*Oryza sativa* L.) is considered to be a healthy food due to its high content of anthocyanins in the pericarp. The synthetic pathway of anthocyanins in black rice grains has been identified, however, the proteomic profile of leaves during grain development is still unclear. Here, isobaric Tags Relative and Absolute Quantification (iTRAQ) MS/MS was carried out to identify statistically significant changes of leaf proteome in the black rice during grain development. Throughout three sequential developmental stages, a total of 3562 proteins were detected and 24 functional proteins were differentially expressed 3–10 days after flowering (DAF). The detected proteins are known to be involved in various biological processes and most of these proteins were related to gene expression regulatory (33.3%), signal transduction (16.7%) and developmental regulation and hormone-like proteins (12.5%). The coordinated changes were consistent with changes in regulatory proteins playing a leading role in leaves during black rice grain development. This indicated that signal transduction between leaves and grains may have an important role in anthocyanin biosynthesis and accumulation during grain development of black rice. In addition, four identified up-regulated proteins associated with starch metabolism suggested that the remobilization of nutrients for starch synthesis plays a potential role in anthocyanin biosynthesis of grain. The mRNA transcription for eight selected proteins was validated with quantitative real-time PCR. Our results explored the proteomics of the coordination between leaf and grain in anthocyanins biosynthesis of grain, which might be regulated by signal transduction and sugar metabolism in black rice leaf.

## 1. Introduction

Rice is one of the most important cereal crops in the world, and white rice is the most commonly consumed type of rice. However, there are several rice cultivars that contain pigments, such as black rice which were popular due to high anthocyanin (ACN) content, which gives it a distinctive dark purple color [1].

Anthocyanins (ACNs) are water-soluble, natural pigments that are responsible for the red, purple and blue colors of many fruits and flowers, which are attractive to pollinators and seed dispersers [2]. Dietary anthocyanins are associated with protection against certain cancers, cardiovascular diseases and other chronic human disorders in addition to their physiological roles in plants [3]. This provides a strong basis for engineering anthocyanin biosynthesis in plants, which would have applications in a diversity of situations. Identification of genes encoding transcription factors that regulate the expression of functional genes and cloning of the key enzyme genes have been reported extensively in plants [4]. Expression of the UDP (uridine diphosphoglucuronate)-glucose flavonoid 3-*O*-glucosyltransferase (*UFGT*) gene has been shown to be critical for anthocyanin biosynthesis in the grape berry [5]. Five anthocyanin biosynthetic genes (chalcone synthase (*CHS*), flavanone-3-hydroxylase (*F3H*), dihydroflavonol 4-reductase (*DFR*), anthocyanidin synthase (*ANS*) and *UFGT*) in apple fruits are coordinately expressed during red coloration of the skin. In addition, the expression levels of these genes have been shown to be positively related to anthocyanin concentrations [6]. Recent studies have indicated that the expression of biosynthetic genes in anthocyanin accumulation is regulated by the MYB (v-myb avian myeloblastosis viral oncogene homolog) transcription factor in the fruit of apples, grapes, Chinese bayberries, mangosteen and red pear [7].

However, in black rice, the regulation of anthocyanins biosynthesis is still far from being understood. There is more available information on anthocyanin concentrations and composition changes during grain development and coloration being improved by spraying of growth regulators [8,9,10]. An iTRAQ-LC-MS/MS (isobaric tag for relative and absolute quantification-liquid chromatograph-mass spectrometer/mass spectrometer) proteomics approach was used in this study to investigate relationship between leaf proteomic and anthocyanin metabolism of black rice grain (*Oryza. sativa* L. *indica* var. subspecies). We compared gene expression patterns to identify candidate factors that could be involved in the regulation of ACN biosynthesis. In addition, some significantly changed proteins were further analyzed to determine whether alterations in protein abundance were coordinated with transcript analysis. Our study provides fundamental information for further investigations of anthocyanin biosynthesis in black rice grains, and facilitates future breeding programs of hybrid varieties of rice with high anthocyanins.

## 2. Results

### 2.1. General Information on iTRAQ Analysis

To study the relationship between leaves and anthocyanin biosynthesis of filling grain in black rice, the proteomics profile of leaves during the grain filling stage was investigated. An iTRAQ proteomics method was applied for comparative analysis of total proteins in leaves of black rice on 3 DAF (day after flowering), 7 DAF, 10 DAF, 15 DAF and 20 DAF. A total of 240,473 MS/MS fragmentation information was detected, and 55,178 MS/MS PSM (protein spectrum matched) could be matched with available database. A total of 10,834 peptides and 3562 proteins were identified based on the matching analysis (Figure S1).

Among the 3562 identified proteins, 3405 proteins had quantitative data for all five sampling time points (3 DAF–20 DAF) (Table S1). The quantitative analysis of most proteins coverage rates was no more than 50% in 3405 quantitative proteins and the number of peptides corresponding to each identified protein was less than 20% (Figure S2A). The isoelectric point and molecular weight of most identified proteins belong to alkaline proteins. In addition, most proteins with molecular weight greater than 100 kDa were identified due to the improved methods for protein extraction and FASP (the Filter Aided Sample Preparation) enzyme digestion (Figure S2B) [11,12].

### 2.2. Comparative Analysis of Protein Expression at Five Developmental Stages

**Figure 1:**
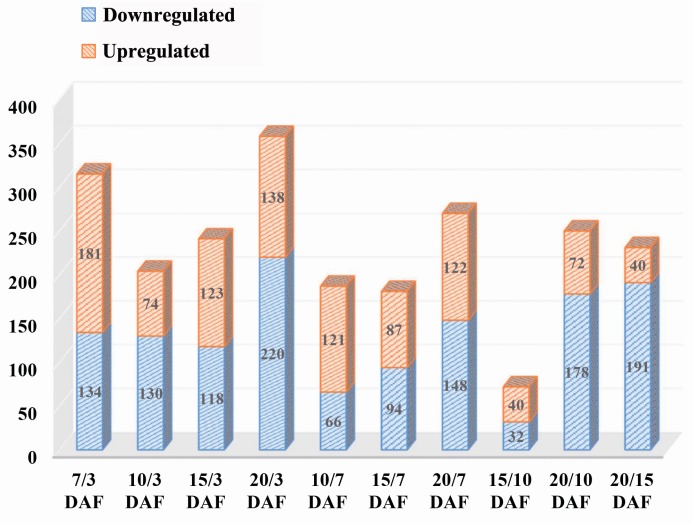
Distribution map of several differentially expressed proteins. A total of 848 proteins with a significant difference between any two time points were detected. The comparison between 20 DAF (day after flowering ) and 3 DAF yielded the most differentially expressed proteins (358), including 138 up-regulated proteins and 220 down-regulated proteins, followed by the comparison between 7 DAF and 3 DAF and the comparison between 20 DAF and 7 DAF. The comparison between 15 DAF and 10 DAF had the least amount of differentially expressed proteins (72), including 40 up-regulated and 32 down-regulated proteins. The X axis represents the comparison between different time points, while the Y axis represents the number of differentially expressed proteins.

Significant differences between any of the five groups were analyzed based on a change greater than 1.5-fold and *p*-value < 0.05. A total of 848 proteins with a significant difference between any two time points were detected. The comparison between 20 DAF and 3 DAF yielded the most differentially expressed proteins (358), including 138 up-regulated proteins and 220 down-regulated proteins, followed by the comparison between 7 DAF and 3 DAF and the comparison between 20 DAF and 7 DAF. The comparison between 15 DAF and 10 DAF had the least amount of differentially expressed proteins (72), including 40 up-regulated and 32 down-regulated proteins (Figure 1). These findings implied that the metabolic activity levels of the leaves was relatively higher at the early and late stages than the middle stage during grain development.

**Figure 2:**
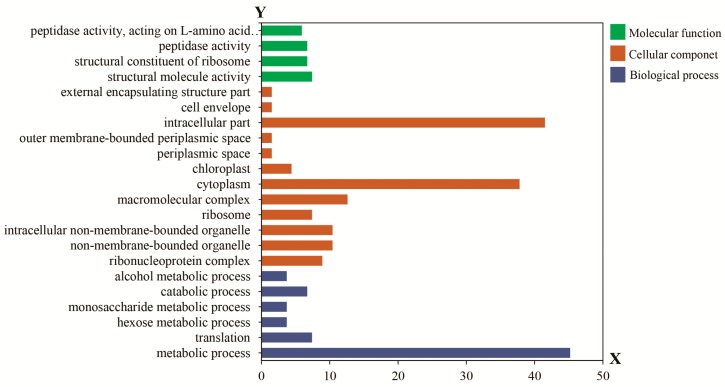
Gene Ontology enrichment analysis results for differentially expressed proteins. The X axis represents the number of differentially expressed proteins; the Y axis represents the Gene Ontology functional classification.

Gene Ontology functional enrichment analysis was performed for the 848 differentially proteins which were classified into four significantly enriched functions: structural constituent of ribosomes, structural molecule activity, and peptidase activity acting on L-amino acid peptides (Figure 2). Subcellular component analysis showed significant enrichment in external encapsulating structure parts, the cell envelope, intracellular parts, outer membrane-bounded periplasmic space, periplasmic space, chloroplasts, cytoplasm, macromolecular complex, ribosomes, intracellular non-membrane-bounded organelles, non-membrane-bounded organelles, and the ribonucleoprotein complex. For biological process analysis, significant enrichment was related to translation, hexose metabolic processes, monosaccharide metabolic processes, catabolic processes and alcohol metabolic processes.

KEGG (Kyoto Encyclopedia of Genes and Genomes) pathway enrichment analysis on the 848 differentially expressed proteins and significant enrichment was divided into five pathways (*p* ≤ 0.05): phenylpropanoid biosynthesis, plant hormone signal transduction, folate biosynthesis, biosynthesis of unsaturated fatty acids, and linoleic acid metabolism (Table 1).

### 2.3. Cluster Analysis of Differentially Expressed Proteins

**Figure 3:**
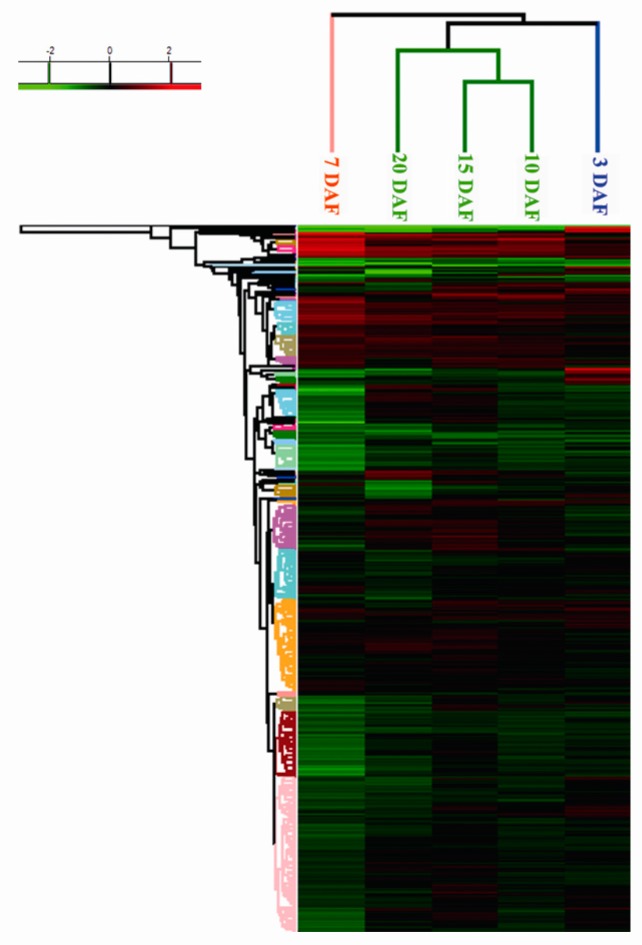
Cluster analysis of differentially expressed proteins in black rice leaves. The five samples with 848 differentially expressed proteins were clustered into three modules. The expression pattern was similar between 10 DAF and 15 DAF, and 15 DAF and 20 DAF. However, pattern in 7 DAF and 3 DAF showed different to that of 10, 15, and 20 DAF, respectively. Green, reduced expression compared to that observed in the REF (reference); Red, increased expression compared to that detected in the REF.

Based on fold-changes in protein expression levels, cluster analysis was performed for proteins differentially expressed in leaves of five developmental stages using Heatmap Clustering software (Unscrambler, Version 10.0.1, Camo Software Inc., Woodbridge, NJ, USA) (Figure 3). The five samples were clustered into three modules. The expression pattern was similar between 10 DAF and 15 DAF, and 15 DAF and 20 DAF. However, pattern in 7 DAF and 3 DAF showed different to that of 10, 15, and 20 DAF, respectively. Anthocyanin measurement in the previous experiment showed that the content of anthocyanins in black grain reached the highest value at 10 DAF [13]. The synthesis of anthocyanins was not only mediated by the expression of structural genes and regulatory genes, but also related to the external environment such as nutrition and energy supplement [14]. The cluster analysis here showed that the protein expression pattern in the leaves on 7 DAF was different from other stages, which implied that the metabolism in leaves of 7 DAF played a different role for the grains development during the grain filling stage.

**Figure 4:**
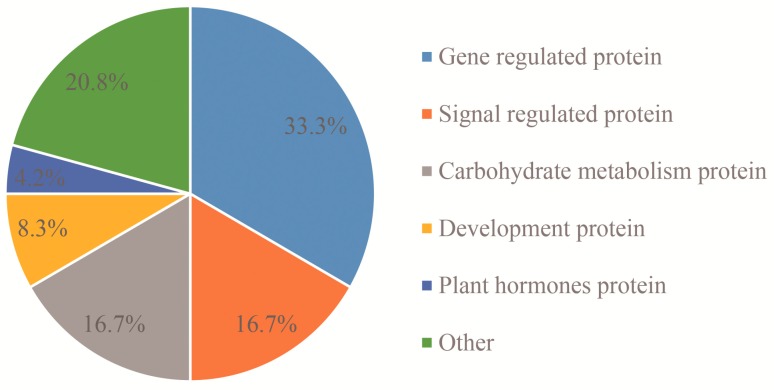
Functional classification of 24 differentially expressed proteins in leaves. Functional classification of these 24 proteins showed that 33.3% were related to gene regulation, 16.7% to signal regulation, 16.7% to carbohydrate metabolism, 8.3% to developmental regulation, 4.2% to plant hormone regulation, and 20.8% to stress regulation and other functions.

Further analysis was performed for leaf proteins on 3 DAF, 7 DAF and 10 DAF and found that 88 differentially expressed proteins were shared between the 7/3 DAF comparison and 10/7 DAF comparison. There were 24 differentially expressed proteins out of 88 with known biological functions, and these proteins could be classified into six functional categories: gene regulated proteins (8, 33.3%), signal regulated proteins (4, 16.7%), carbohydrate metabolism-related proteins (4, 16.7%), development proteins (2, 8.3%), phytohormone protein (1, 4.2%) and others (5, 20.8%) (Figure 4).

Gene regulated proteins were illustrated as follows: nucleic acid binding protein 1 (Nabp1, AAD31844.1), cysteine-rich receptor-like protein kinase 10 (CRK10, XP_015645459.1), RNA polymerase A(I) large subunit (RPS, BAD35511.1), CRM (chloroplast RNA splicing and ribosome maturation )-domain containing factor CFM3 (CFM3, Q2R1U8.1), ribosomal protein L32 (RPL32, AER12970.1), retrotransposon protein (REP, ABF98360.1), putative ubiquitin/ribosomal protein CEP52 (carboxyl-extension protein 52) (CEP52, AAM19122.1), polyadenylate-binding protein 1 (PABPN1, XP_015635554.1). There were four kinds of signal regulated proteins identified: phytochrome B isoform X1 (PhyB, XP_015631281.1), F-box protein At5g18160-like (FP, XP_015647449.1), mitogen-activated protein kinase 1 isoform X1 (MAP2K1, XP_015621392.1), histone deacetylase complex subunit SAP18 (Sin3-associated polypeptide 18) (HDAC, XP_015626312.1). Fructose-bisphosphate aldolase (FBAP, ABA91632.2), beta-galactosidase (β-gal, XP_015620214.1), phosphoglycerate kinase (PK, XP_015644193.1), probable galactinol-sucrose galactosyltransferase 2 isoform X1 (RFS2, XP_015620380.1) were carbohydrate metabolism proteins, while 60 kDa jasmonate-induced protein-like (JIP60, XP_015616478.1) was phytohormone protein. Two proteins related to the development were found: anaphase-promoting complex subunit 8-like protein (ANAPC8, CAC39070.1) and probable linoleate 9S-lipoxygenase 4 isoform X1 (PLL4, XP_015632132.1). Five other proteins were discovered: disease resistance RPP13-like protein 3 (RPP13, XP_015626169.1), putative gag-pol polyprotein (Gag-pol, AAO17007.1), MA3 domain-containing protein (MA3, BAD10818.1), stemar-13-ene synthase (XP_015624477.1), and expressed protein (AAT77840.1).

### 2.4. Validation Using Quantitative RT-PCR

**Figure 5:**
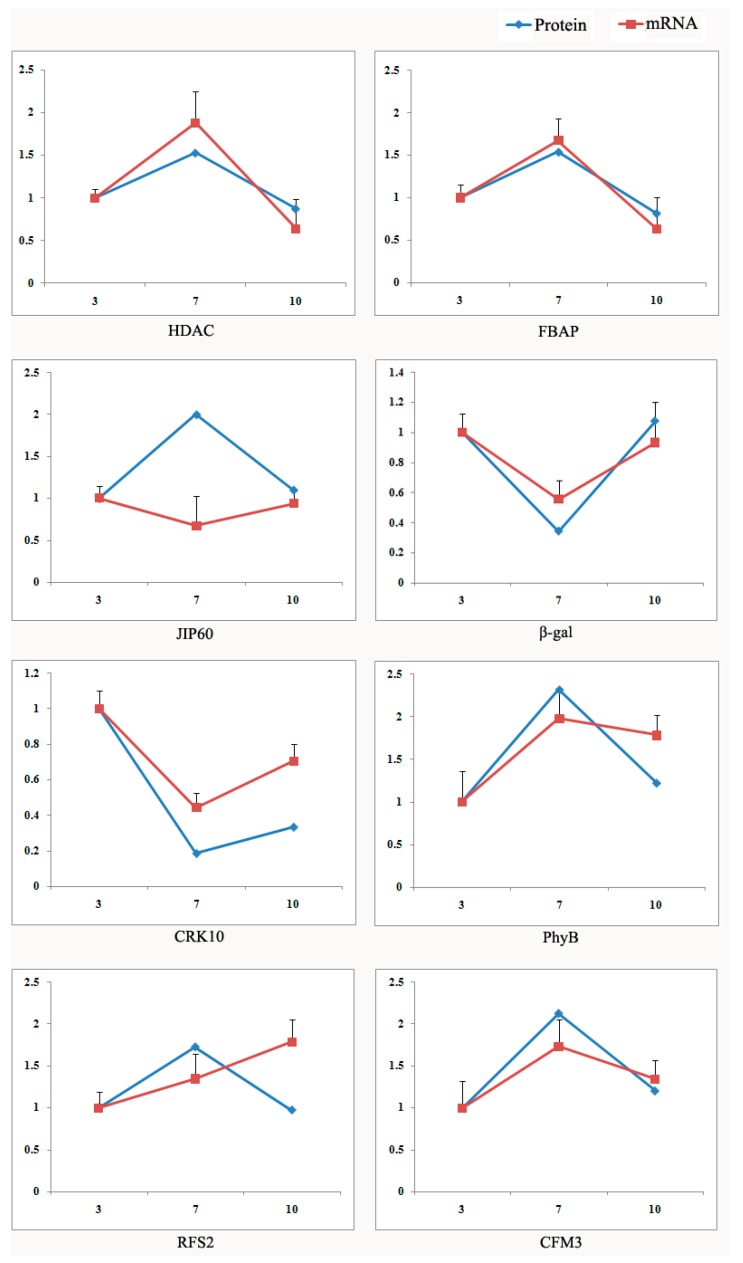
The expression trends in mRNA and protein levels of eight differentially expressed proteins. To assess the mRNA transcription profiles of the 24 functional proteins, eight genes of them were selected and verified by quantitative RT-PCR (qRT-PCR): the histone deacetylase complex subunit SAP18 (*HDAC*), fructose-bisphosphate aldolase (*FBAP*), 60 kDa jasmonate-induced protein-like (*JIP60*), beta-galactosidase (*β-gal*), cysteine-rich receptor-like protein kinase 10 (*CRK10*), phytochrome B isoform X1 (*PhyB*), galactinol—sucrose galactosyltransferase 2 isoform X1 (*RFS2*), and CRM-domain containing factor CFM3 (*CFM3*). There was a similar pattern between mRNA expression and protein synthesis for HDAC, FBAP, β-gal, CRK10, PhyB and CFM3; However, JIP60 and RFS2 showed different trend in these two profiles. The X axis represents the different days after flowering (DAF), while the Y axis indicates the relative expression level of protein and mRNA. The blue line represents the pattern of protein expression, and the red line indicates the pattern of mRNA expression.

To assess the mRNA transcription profiles of the 24 functional proteins, eight genes were selected and verified by quantitative RT-PCR (qRT-PCR): the histone deacetylase complex subunit SAP18 (*HDAC*), fructose-bisphosphate aldolase (*FBAP*), 60 kDa jasmonate-induced protein-like (*JIP60*), beta-galactosidase (*β-gal*), cysteine-rich receptor-like protein kinase 10 (*CRK10*), phytochrome B isoform X1 (*PhyB*), galactinol—sucrose galactosyltransferase 2 isoform X1 (*RFS2*), and CRM-domain containing factor CFM3 (*CFM3*). There was a similar pattern between mRNA expression and protein synthesis for HDAC, FBAP, β-gal, CRK10, PhyB and CFM3 (Figure 5). However, JIP60 and RFS2 showed different trend in these two profiles. Increasing evidence shows that the expression abundance of mRNAs does not necessarily match their corresponding proteins because gene expression is influenced by many factors, not only transcriptional regulation, but also post-transcriptional regulation, translation and post-translational processing and modification. In addition, mRNA degradation, protein degradation, folding and modification may lead to inconsistent mRNA abundance and protein expression level [15,16]. We deduced the difference between mRNA and protein levels of JIP60 and RFS2 might be related to translational or post-translational modifications [17,18].

## 3. Discussion

During the process of carbon metabolism, the accumulation of sugars has an important role in the biosynthesis of starch and anthocyanins of black rice grains [19,20,21]. To further understand the metabolic regulation network of anthocyanin synthesis in developing grains, proteomic analysis was performed on leave which was the source of sugar supplement. Since the leaf protein expression pattern on 7 DAF was different from the other four time points (3 DAF, 10 DAF, 15 DAF and 20 DAF), and, considering that the results of previous studies showed that anthocyanin content reached a maximum on 10 DAF [13], we hypothesized that the unique protein expression pattern of leaves on 7 DAF might have potential connection with the high expression of 10 DAF in anthocyanin of grain.

### 3.1. Signal Regulation-Related Proteins

**Figure 6:**
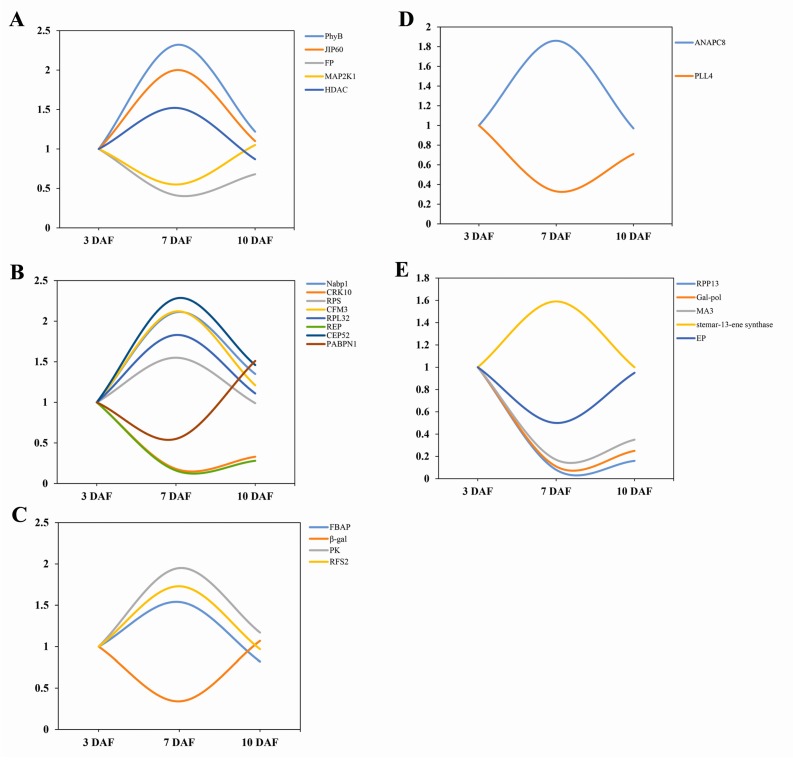
The expression of different functional proteins in leaves at different developmental stages: (**A**) signal regulated proteins and plant hormone proteins; (**B**) gene expression regulatory proteins; (**C**) carbohydrate metabolism-related proteins; (**D**) development-associated proteins; and (**E**) other proteins (the X axis represents the time point of kernel development, while the Y axis represents the relative expression level of proteins on 3 DAF).

According to previous research, light enhanced the accumulation of pigment and induced the expression of R2R3-MYB, a transcription factor in the anthocyanin biosynthetic pathway [22,23]. Some researchers also reported that phytochrome activated by light promoted the synthesis or activity of related enzymes [24,25,26]. PAL (phenylalanine ammonia-lyase), CHS, CHI (chalcone isomerase), DFR, ANS, UFGT and MYB regulatory protein involved in anthocyanin biosynthesis are light-regulated enzymes [27,28,29], and therefore they can be activated by light and thus contribute to anthocyanin accumulation as well as the pigmentation of the fruit [30,31,32]. In our research, we found that phytochrome B (PhyB) subtype X1 expression in leaves of black rice had a highest abundance after seven days of flowering (Figure 6A). It is speculated that it might regulate metabolic activities such as the synthesis of anthocyanins through photomorphogenesis [33].

In response to the light signal, plants regulate the expression of related genes through a cascade of reactions. The members of F-box protein family played a role for substrate recognition in ubiquitin-mediated protein degradation [34]. F-box proteins performed important functions in plant hormone signal transduction, light signal transduction, self-incompatibility and flower organ development, and many other physiological processes [35].

Mitogen-activated protein kinases (MAPKs) are a kind of intracellular serine/threonine protein kinases [36], and the MAPK signaling pathway mediates a variety of important cellular physiological responses. Bolouri and Menelink reported that MAPK kinase activity is related to the anthocyanin metabolism [37,38]. MAPK is composed of three kinds of protein kinase—MAP3K, MAP2K, and MAPK—and the upstream signal is transmitted to downstream effectors by phosphorylation [39]. This study found that MAP2K1, one of the MAPK members, showed clear down-regulation on 7 DAF (Figure 6A); therefore, we speculated that it may be related to the accumulation of anthocyanins in the same period.

Histone deacetylase (HDAC) is a protease playing an important role in the structural modification and regulation of gene expression [40]. Currently, most works have indicated that high expression of HDAC improved the plants adaptability to stress [41]. Kim et al. found that, in *Arabidopsis thaliana*, HDAC can promote the expression of anthocyanin through abscisic acid [42]. In the present study, HADC showed the highest expression levels on 7 DAF (Figure 6A), which was consistent with high expression of HADC being able to promote the synthesis of anthocyanins.

### 3.2. Phytohormone Proteins

Jasmonate, a class of important growth regulators, is an environmental signal molecule which has significance in multiple physiological processes of plants [43]. Jasmonate promoted the absorption of nitrogen and phosphorus and involved in the transport of organic compounds such as glucose. Jasmonate especially mediates defense responses to biotic and abiotic stresses, as it creates an emergency response to pathogen infection and induces gene expression by signal transduction [44]. Jasmonic acid promoting protein (60 kDa; JIP60) was also identified to have the highest expression level on 7 DAF (Figure 6A). Some studies have found that high expression of jasminic acid in grape and rubber tree promoted the synthesis of anthocyanins [45,46,47].

### 3.3. Gene Expression Regulatory Proteins

Plant cells can respond to biotic and abiotic stimulus and regulate gene expression through the signal transduction at the transcriptional, post-transcriptional, translational and post-translational levels in plant cells. NBP (nucleic acid binding protein) can bind DNA and RNA, and can regulate gene expression in transcriptional level [48]. Sainz et al. found that enhanced expression of NBP in potato can activate anthocyanin synthesis activator [49]. Mathews et al. studied transcription regulation in photosynthesis and discovered that NBP is involved in the synthesis, modification and transport of anthocyanins [50]. In the present study, we found two proteins with NBP properties: nucleic acid binding protein 1 (Nabp1) and polyadenylate-binding protein 1 (PABPN1). Expression level of Nabp1 was increased by 2.11 fold on 7 DAF/3 DAF, but was down-regulated by 1.56 fold on 10 DAF/7 DAF (Figure 6B). Highest Nabp1 expression was detected on 7 DAF, while the lowest PABPN1 level was observed on 7 DAF (Figure 6B). The different expression patterns of Nabp1 and PABPN1 may contribute to the levels of intracellular NBP, thereby promoting the synthesis of anthocyanins and enhancing the transport of anthocyanins to grains. In addition, studies have shown that, in *Arabidopsis*, NBP and cysteine-rich receptor-like protein kinase 10 (CRK10) synergistically prevent pathogen infection and salicylic acid stress [51]. However, the synergism between CRK10 and other NBPs (Nabp1 and PABPN1) and the role on promoting effect of anthocyanin synthesis remains to be further investigated.

RNA polymerase catalyzed the synthesis of RNA from nucleoside 5’-triphosphate with single DNA or RNA chains as templates [52,53]. Quattrocchio et al. found that plant regulatory genes can be controlled by RNA polymerase to control the accumulation of anthocyanins [54]. A study by Zhou et al. showed that abscisic acid in *Arabidopsis thaliana* regulate anthocyanin synthesis by RNA polymerase [55]. Our results showed that RNA polymerase A (I) large subunit (RPS) reached the highest expression on 7 DAF (Figure 6B), so we speculated would produce a positive effect on the accumulation and synthesis of anthocyanins.

The CRM functional domain binding factor CFM3 was participate in the alternative splicing of DNA transcription and expression of alternative mRNAs [56]. The poly(A)-binding protein (PABP) is a highly conserved protein that binds the Poly(A) tail of mRNA and regulates mRNA translation and stability. The regulations between CFM3 and PABP still to be answered.

Ribosomal protein L32 (RPL32) includes 80 different subtypes that are widely distributed in various tissues [57], and plays an important role in the biosynthesis of proteins [58]. Some research indicated that RPL32 has a negative effect on the accumulation of anthocyanins [59]; however, our results showed that RPL32 reached the highest expression on 7 DAF. The regulation of ribosomal protein is extensive but not specific, so the specific regulation pattern of RPL32 in the leaf remains to be further studied.

The Ubiquitin proteasome pathway is the main pathway for protein degradation in eukaryotic cells [60]. CEP52 is an important enzyme in the ubiquitin proteasome pathway. Research has shown that CEP52 constitutively photomorphogenic 1 (COP1) is a repressor of photomorphogenesis downstream of light receptors and serves as a molecular switch for light-regulated plant development [61]. COP1 inhibits the response to light under dark conditions. The COP1/SPA (suppressor of phyA) complex regulates the expression of structural genes in anthocyanin synthesis by interacting with MYB regulatory factors, thus affected the synthesis of anthocyanins [62,63]. We speculate that the high expression of CEP52 on 7 DAF may be related to the synthesis of anthocyanins (Figure 6B).

Plant retrotransposon is widely distributed as a class of mobile genetic factors in the plant [64]. Many studies have shown that most retrotransposons can be activated by stresses such as protoplast isolation, chilling injury, virus, bacterial inoculation, and so on. Retrotransposons play important roles in the structure, function and evolution of plant genomes [65]. Butelli et al. found that, in blood oranges, insertion of Copia-like retrotransposon regulates the activity of the MYB transcription activator Ruby, which regulates anthocyanin synthesis [66]. In our study, retrotransposon protein (REP) showed the lowest expression level on 7 DAF due to the partial feedback regulation mechanism of retrotransposons (Figure 6B). Therefore, the specific function and expression patterns of REP in the leaves need to be further studied and analyzed.

### 3.4. Carbohydrate Metabolism-Related Proteins

During grain-filling stage, the main physiological role of the leaves is to provide C and N metabolites that are transported to the grains to make seed reservoirs. N metabolism mainly affects the contents of amino acids, proteins and nucleic acids, and the metabolism of organic compounds such as phospholipids, chlorophyll and vitamins in rice. In the experiment, we found four differentially expressed proteins closely related to N metabolism: glutamate formimidoyltransferase-like (XP_015642890.1), glutathione transferase GST 23 (XP_015611712.1), glutathione S-transferase zeta class isoform X1 (XP_015625161.1) and putative glutaminyl-tRNA synthetase (AAU44312.1).

Plant carbon metabolism is a complex process involving tricarboxylic acid cycle (TCA), glycolysis/photorespiration, photosynthesis, and so on. Carbohydrates have an important role on the metabolism, growth and development of plants [67]. During grain-filling stage, 19 differentially expressed proteins closely related to C metabolism were identified at any two time points. According to expression pattern of proteins between adjacent time points (DAF7/3, DAF10/7, DAF15/10, DAF20/15), we divided the differentially expressed proteins into three classes.

Firstly, four proteins significantly changed in both DAF7/3 and DAF10/7: FBAP, β-gal, PK, and RFS2 (Figure 6C). FBAP is one of the most important isozymes involved in primary metabolic responses in plants [68]. It catalyzes the aldol reaction of dihydroxyacetone phosphate and glyceraldehyde 3-phosphate and this generates 1,6 fructose diphosphate and is involved in gluconeogenesis and glycolysis. It is also involved in the pentose phosphate pathway and Calvin cycle [69]. The β-galactosidase (full name β-D-glucoside hydrolase), is a versatile enzyme that is widely distributed in various plants and animals. This enzyme can hydrolyze lactose and also showed galactosyltransferase activity. RFS2 in plants has similar functions with β-galactosidase, participating in a series of physiological and biochemical processes, such as the pollen development of plants and the cleavage of polysaccharides during fruit ripening. PK is the key enzyme of fermentation, and is an enzyme that is necessary for the survival of each organism. A deficiency of the enzyme can cause disorders of metabolism. Most organisms contain 2–3 kinds of PK isozymes that present with different distributions and biological functions.

Secondly, nine proteins significantly changed only in DAF7/3 or DAF10/7, and fructose-bisphosphate aldolase class-I (AAX95075.1) changed significantly in both the early and the late grain-filling stages (DAF7/3 and DAF 20/15). Seven proteins which contained 1,3-beta-glucan synthase component-like (BAC01168.1), UDP-glucose 6-dehydrogenase 4 (XP_015620155.1), divinyl chlorophyllide a 8-vinyl-reductase (D5L1S4.1), phosphoenolpyruvate carboxylase 2 (XP_015611998.1), glucan endo-1,3-beta-glucosidase GII (XP_015622443.1), probable glucan 1,3-alpha-glucosidase (XP_015631638.1), probable acetyltransferase NSI (nuclear shuttle protein) (A2Y5T7.2) were differentially expressed only in DAF7/3; while two proteins changed significantly only in DAF10/7: putative triose phosphate/phosphate translocator (BAB17213.1) and 2-oxoglutarate/malate translocator (ABA98704.1). These proteins are involved actively in tricarboxylic acid cycle, glyoxylate cycle, glycolysis and participate in the metabolism of nutrients such as starch, sucrose, porphyrins and chlorophyll.

Finally, some proteins did not change significantly between adjacent time points, but they express differentially during a period of time (e.g., DAF20/3, DAF15/7, and DAF20/7). Ribulose 1,5-bisphosphate carboxylase (CAD29282.1) and ribulose-1,5-bisphosphate carboxylase/oxygenase large subunit (ADD62840.1) are important carboxylases in photosynthesis, and also indispensable oxygenases in photorespiration. Glyceraldehyde-3-phosphate dehydrogenase (AAB66887.1) is the key link of glucose metabolism, while cytochrome P450 84A1 (ABG66184.1) and photosystem I reaction center subunit XI (XP_015620442.1) are important components of chloroplast and essential for photosynthesis in plants.

These 19 differentially expressed proteins with different expression patterns may contribute to balance C metabolism in plants, and enhanced the synthesis of carbohydrates. In the previous study, we found photosynthesis showed a decreased tendency of expression in black rice grains during filling stage which would limit the synthesis of sugars in grains [13]. As sugars are precursors to the synthesis of starch and anthocyanins in grains, the leaves which are the largest source of plants would play an essential role in carbohydrate synthesis and transportation. The sugars in the leaves can be transported to the grain via the phloem. Fifty percent of the sugar-related organic compounds in wheat grains were derived from the flag leaf of the spike. Glucose transported from flag leaf into spikes is synthesized in photosynthesis. Studies have shown that carbohydrates can significantly promote the synthesis of anthocyanins as well as other carbohydrates besides fructose, glucose, and sucrose, including maltose, raffinose, trehalose, cellobiose, melibiose, lactose, and galactose. We speculate that carbohydrates in leaves during the grain filling stage of black rice can be transported to the grain via phloem, where it can be used as raw materials for the biosynthesis of starch and carbohydrates necessary for anthocyanin synthesis, as well as signal molecules that control anthocyanin synthesis in grains [70,71,72].

### 3.5. Development-Associated Protein

Plant morphogenesis is the result of the selective expression of the genes with a specific temporal and spatial pattern. Different stages of differentiation will synthesize different proteins [73]. ANAPC is closely related to cell mitosis and meiosis and encoded an E3 ubiquitin ligase. Pineiro et al. summarized that ANAPC regulates the photoperiod to control the flowering time of plants, and the different time of flowering will have different effects on the synthesis of anthocyanins [74]. In our study, we detected that ANAPC8 was up-regulated on 7 DAF/3 DAF by 1.82 folds (Figure 6D), which implied the physiological function of leaves during the grain filling stage is regulated by the photoperiod and flowering signals. PLL4 is not well studied. According to its amino acid sequence, it is speculated that it may be involved in many aspects of plant physiology, such as growth, disease and pest protection, as well as aging [75]. In this study, we observed low expression of PLL4 on 7 DAF, which may be related to its specific physiological function and this needs to be further investigations.

### 3.6. Other Proteins

Five other proteins have been discovered, including RPP13, Gag-pol, MA3, stemar-13-ene synthase, and expressed protein (Figure 6E). In the process of growth, plants will encounter stress such as high (low) temperature, drought, water logging and high salt and other abiotic stress, and biotic stresses such as pathogen infections and pests. Upon sensing of the stress signal, plants adjust their physiological state or morphology by regulating of stress-associated proteins level and and improve tolerance to stress. Plant stress proteins may be derived from the accumulation induced by stress, while it can also play roles in gene regulation [76]. In the present study, the up-regulated expression of the disease resistance protein RPP13 maybe not only a simple accumulation, but also having a regulatory role in plant defense. However, few studies have been carried out on Gag-pol, MA3, stemar-13-ene synthase and expressed protein, and the impact on leaf growth remains to be further studied.

## 4. Materials and Methods

### 4.1. Plant Materials and Sampling

Black rice (*Oryza. sativa* L. *indica* var. subspecies) plants were cultivated from May to September under natural conditions at the Fujian Agriculture University experimental research fields and were fertilized (urea, 60 kg/ha) with practical procedures. In previous studies, according to the changes of anthocyanin content at grain filling stage, seeds from 3 days after flowering (DAF), 7 DAF, 10 DAF, 15 DAF and 20 DAF were selected for proteomics research [13]. In this experiment, for 3 DAF, 7 DAF, 10 DAF, 15 DAF and 20 DAF, the topmost three leaves were cut from the tillers of the collected seeds. The leaves were cleaned with deionized water, then wrapped in aluminum foil and stored at −80 °C.

### 4.2. Protein Extraction and Digestion

One gram of frozen mixed leaves from six replications was ground in liquid nitrogen. The ground leaves were then precipitated in 25 mL of ice-cold trichloroacetic acid-acetone (10% trichloroacetic acid mixed in 100% acetone) for 4 h at −20 °C. This was followed by centrifugation at 35,000× *g* for 20 min for isolation. Cold 80% acetone with 0.07% (*w*/*v*) β-mercaptoethanol was used to wash the protein pellet first, then this was followed by cold acetone with 0.07% (*w*/*v*) β-mercaptoethanol [77]. The homogenate was centrifuged and the pellets were air-dried. They were then mixed in 800 µL STD buffer (4% SDS, 150 mM Tris-HCl, 100 mM DTT, pH 7.6), boiled for 5 min, and then sonicated. After centrifugation, the supernatants were then collected, and the supernatants were collected after centrifugation. The protein content was measured with a BCA protein assay reagent (Beyotime Institute of Biotechnology, Jiangsu, China). Equivalent amounts of proteins (120 µg from 3 DAF, 7 DAF, 10 DAF, 15 DAF, and 20 DAF) were then mixed and used as a reference (REF).

The FASP procedure was used for protein digestion [78]. A total of 300 µg of proteins from each sample were placed on an ultrafiltration filter (30 kDa cut-off, Sartorius, Gottingen, Germany) that had 200 µL of UA buffer (8 M urea, 150 mM Tris-HCl, pH 8.0). It was then centrifuged at 14,000× *g* for 30 min and then washed with 200 µL of UA buffer. About 100 µL of 50 mM iodoacetamide was added to the filter to block any reduced cysteine residues. The samples were maintained at room temperature for 30 min in the dark. This was followed by centrifugation at a speed of 14,000× *g* for 30 min. UA buffer (100 µL) was used to wash the filters twice. This was followed by centrifugation at 14,000× *g* for 20 min after every wash. Approximately 100 µL of a dissolution buffer (Applied Biosystems, Foster City, CA, USA) was placed on the filter. This was centrifuged at 14,000× *g* for 20 min, and then repeated twice. The protein suspensions were subjected to enzyme digestion with 40 µL of trypsin (Promega, Madison, WI, USA) buffer (4 µg trypsin in 40 µL of dissolution buffer) for 16–18 h at 37 °C. The final filter unit was transferred to a new tube that was spun at 14,000× *g* for 30 min. The peptides were collected as a filtrate and the concentration of the peptides was measured at an optical density with a 280 nm wavelength (OD_280_).

### 4.3. iTRAQ Labeling and Strong Cation Exchange

iTRAQ labeling was conducted according to the manufacturer’s recommendations (Applied Biosystems, Foster City, CA, USA). Briefly, the peptide pellet was reconstituted in 30 µL of the iTRAQ dissolution buffer. The iTRAQ Reagent-8plex Multiplex Kit (AB SCIEX, Framingham, MA, USA) was used to label each sample (100 µg) twice at 3 DAF, 7 DAF, 10 DAF, 15 DAF, and 20 DAF. Each group then received a REF sample. This labeling reaction was maintained for 1 h at room temperature and was then analyzed. Six labeled samples from each group were pooled and then vacuum-dried in a centrifuge at room temperature.

Strong cation exchange fractionation with an AKTA Purifier 100 (EG Healthcare, Little Chalfont, Bucks, UK), which had a polysulfethyl (PolyLC Inc., Columbia, MD, USA) column (4.6 mm × 100 mm, 5 µm, and 200 Å), was used on the iTRAQ-labeled peptides. Elution of the peptides was then performed at a flow rate of 1 mL/min. Buffer A was made up of 10 mM KH_2_PO_4_ and 25% *v*/*v* can (pH 3.0), whereas Buffer B contained 10 mM KH_2_PO_4_, 25% *v*/*v* ACN and 500 mM KCl (pH 3.0). Both buffers were filter-sterilized. The gradient utilized for separation was the following: 100% Buffer A for 25 min, 0–10% Buffer B for 7 min, 10%–20% Buffer B for 10 min, 20%–45% Buffer B for 5 min, 45%–100% Buffer B for 5 min, 100% Buffer B for 8 min, and lastly, 100% Buffer A for 15 min. The elution process was monitored by measuring the absorbances at a wavelength of 214 nm. A total of 30 fractions were collected at 1 min intervals and then combined into 10 pools. They were then desalted using C18 cartridges (Sigma, Santa Clara, CA, USA). Vacuum centrifugation was used to concentrate every fraction. This was followed by reconstitution in 40 µL of 0.1% (*v*/*v*) trifluoroacetic acid. The samples were maintained at −80 °C until LC-MS/MS analysis.

### 4.4. LC-MSMS Analysis

The Easy-nLC nanoflow HPLC system (Thermo Fisher Scientific, Karlsruhe, BW, Germany), connected to an LTQ Orbitrap Elite mass spectrometer (Thermo Fisher Scientific, Karlsruhe, BW, Germany), was used to analyze the iTRAQ-labeled samples. An autosampler was used to load 1 µg of every sample onto a Thermo Scientific EASY column (two columns) with a flow rate of 150 nL/min. The Thermo Scientific EASY trap column (100 µm × 2 cm, 5 µm, 100 Å, C18) and analytical column (75 µm × 25 cm, 5 µm, 100 Å, C18) was used to conduct peptide sequential separation with a segmented 2-h gradient of Solvent A (0.1% formic acid in water) to 35% Solvent B (0.1% formic acid in 100% ACN) for 100 min. This was then followed by 35–90% Solvent B for 3 min, and finally, 90% Solvent B for 5 min. The column was then re-equilibrated to achieve its initial highly aqueous solvent composition before analysis.

The mass spectrometer was run in a positive ion mode. The MS spectra were obtained in the range 300–2000 *m*/*z*. The resolution of the MS and MS/MS scan was set to 60,000 and 15,000, respectively, using 200 *m*/*z* for the LTQ Orbitrap Elite. The 10 signals with the highest intensities in the acquired MS spectra were chosen for additional MS/MS analysis. The isolation window was 1 *m*/*z*, with ions fragmented using higher energy collisional dissociation at normalized collision energies of 35 eV. The highest ion injection times were 50 ms for the survey scan and 150 ms for the MS/MS scans. The automatic gain control target value for the full scan modes were set to 1.0 × 10^−6^, and the target value for MS/MS was 5.0 × 10^4^. The duration of the dynamic exclusion was 30 s.

### 4.5. Database Search and Protein Quantification

The raw files were analyzed with Proteome Discoverer 1.4 (Thermo Fisher Scientific, Karlsruhe, BW, Germany). Identification of the fragmentation spectra was performed with the MASCOT 2.2 (Matrix Science Inc., Boston, MA, USA) search engine that was embedded in Proteome Discoverer and run against the rice protein database (released in May 2016 and including 144,386 sequences from NCBI). The search parameters were the following: trypsin utilized as the cleavage enzyme, monoisotopic mass, iTRAQ labeling, two missed cleavages, and carbamidomethylation of cysteine used as fixed modifications. The peptide charges of 2+, 3+, and 4+ and methionine oxidation were designated as variable modifications. The mass tolerance was 0.05 Da for fragmented ions and 10 ppm for precursor ions. A false discovery rate (FDR) of <1% was used to filter the results.

We conducted relative quantitative protein analysis of samples using the ratios of iTRAQ reporter ions derived from all unique peptides that represented each protein using Proteome Discoverer software (version 1.4). We used the relative peak intensities of the iTRAQ reporter ions that were derived from each of the MS/MS spectra and the REF sample was the reference sample for calculating the iTRAQ ratios of the reporter ions. The median protein quantification ratio was used to normalize the final ratios derived from the relative protein quantifications. The protein ratios represented the median of the unique peptides in the protein and only proteins identified in both replicates were considered for further analysis.

### 4.6. Bioinformatics Functional Analysis

Perseus V1.4.1.3 (http://141.61.102.17/perseus_doku/) was used to perform statistical and hierarchical clustering analyses [79]. Significance was set at *p*-values < 0.05 by Benjamini–Hochberg FDR in Perseus and a ratio fold-change of >1.50 or <0.67 in expression between any two groups. The following settings were used in hierarchical clustering analysis: Row, Column distance that was calculated using the Euclidean algorithm; Row, Column linkage–Complete. The proteins were categorized according to molecular function, biological process and cellular localization. The differentially expressed proteins were then matched to the Clusters of Orthologous Groups of proteins (COG) database (http://www.ncbi.nlm.nih.gov/COG/).

### 4.7. RNA Extraction and qRT-PCR Analysis

The TaKaRa RNAiso reagent (TaKaRa Bio, Otsu, Japan) was used to extract the total RNA from leaves. The RNA was then treated with RNase-free DNase I (TaKaRa Bio, Otsu, Japan). Reverse-transcription with the M-MLV (Moloney murine leukemia virus) reverse transcription system (Promega, Madison, WI, USA) was then performed on the purified RNA, according to the manufacturer’s instructions. The primers were designed with primer premier 5.0 software [80], and the sequences were listed in Table S2. The CFX96 Real-time System (BioRad, Hercules, CA, USA) was used for qRT-PCR, run in triplicate, that was conducted in 96-well blocks with SYBR Green I master mix in a volume of 25 µL. Samples for qRT-PCR were run in 6 biological replicates with 3 technical replicates. *ACTIN* (GenBank Accession Number: AY212324) was used as an internal reference to normalize the expression data. The 2^−∆∆*C*t^ (cycle threshold) method was used to calculate relative expression levels.

## 5. Conclusions

**Figure 7:**
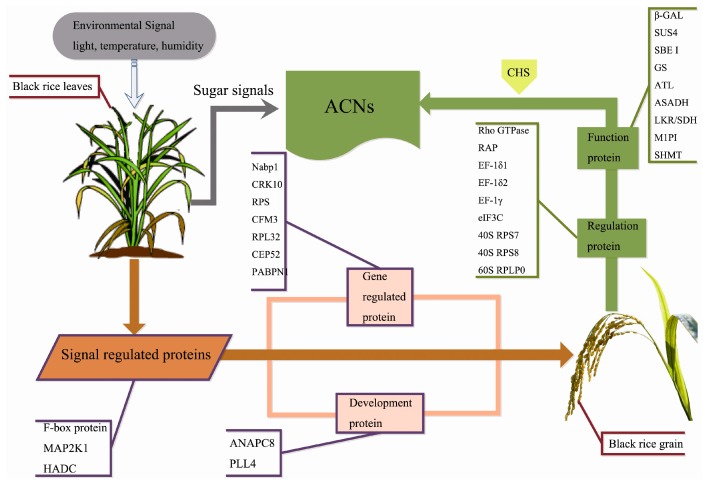
Molecular regulation mechanism of anthocyanin synthesis in the grain filling stage of black rice. In the early stage of grain filling, the regulatory proteins were in an active state of expression in leaf. Signal regulation proteins convert external light, temperature and humidity into intracellular signals that facilitate the expression of proteins that regulate gene expression and development in leaf. The accumulation of these proteins promotes the expression of anthocyanin functional and regulatory proteins, and finally enhances the synthesis of anthocyanin in grains. ACNs, Anthocyanins; Rho GTPase, Mitochondrial Rho GTPase; RAP, Ras-related protein RIC2; EF-1δ1, Elongation factors 1-delta 1; EF-1δ2, Elongation factors 1-delta 2; EF-1γ, Elongation factors 1-gamma; eIF3C, eukaryotic translation initiation factor 3 subunit C; 40S RPS7, 40S ribosomal protein S7; 40S RPS8, 40S ribosomal protein S8; 60S RPLP0, 60S acidic ribosomal protein P0; β-GAL, beta-galactosidase; SUS4, Sucrose synthase 4; SBEI, Starch-branching enzyme I; GS, Glutamine synthetase; ALT, Alanine aminotransferase; ASADH, Aspartate-semialdehyde dehydrogenase family protein; LKR/SDH, Putative lysine-ketoglutarate reductase/saccharopine dehydrogenase bifunctional enzyme; M1PI, Methylthioribose-1-phosphate isomerase; SHMT, Serine hydroxymethyltransferase.

Anthocyanin synthesis involves the expression of various structural genes and regulatory genes, and also relies on the nutrition and energy supply. The accumulation of sugars in the process of carbon metabolism has an important role on the biosynthesis of starch and anthocyanins. To understand the metabolic regulatory network of anthocyanin biosynthesis in developing grains and leaves, which is the largest source of sugar, we analyzed the protein composition of the leaves during the grain filling stage by iTRAQ proteomic analysis, and our results showed that the protein expression pattern on 7 DAF was different from the other four time points (3 DAF, 10 DAF, 15 DAF and 20 DAF). Because anthocyanin content was the highest in grains on 10 DAF [13], we specifically analyzed the protein contents at the following three stages: 3 DAF, 7 DAF and 10 DAF. Twenty-four differentially expressed proteins with known functions were identified, including 33.3% gene expression regulatory proteins, 16.7% signal regulatory proteins, and 12.5% regulatory proteins and hormones. These proteins accounted for more than 60% of the total differentially expressed proteins, indicating that the expression of specific regulatory proteins in the leaves at early stage may promote the synthesis of anthocyanins. Meanwhile, four proteins related to glycometabolism were identified, including FBAP, β-gal, PK and RFS2, which was consistent with previous findings. Leaves, as the major energy source, produce carbohydrates by photosynthesis, which can be transported to grain through the phloem and thus helps to promote the synthesis of starch and anthocyanin. Finally, JIP60 was identified, which greatly promotes the synthesis of JA. JIP60 promotion of anthocyanin synthesis has also been reported by multiple other studies, which is consistent with our data. In summary, combining the results of the present study and previous studies on grains [13], we elucidated the synthesis of anthocyanin and the regulation mechanism of anthocyanin synthesis by proteins in leaf of black rice during the grain filling stage (Figure 7). We speculated that, in the early stage of grain filling, the regulatory proteins were in an active state of expression in leaf. Signal regulation proteins convert external light, temperature and humidity into intracellular signals that facilitate the expression of proteins that regulate gene expression and development in leaf. The accumulation of these proteins promotes the expression of anthocyanin functional and regulatory proteins, and finally enhances the synthesis of anthocyanin in grains. This regulating signal pathway is parallel to the leaf carbohydrate metabolism process. Carbohydrates from leaves are transferred to grains and sugar signals in leaves possible play a role in the regulation of anthocyanin accumulation in black rice grain.

## Figures and Tables

**Table 1:** KEGG (Kyoto Encyclopedia of Genes and Genomes) enrichment analysis results of differentially expressed proteins.

Map ID	Map Name	Diffs	Refs	*p*-Value
ko00940	Phenylpropanoid biosynthesis	21	39	9.28 × 10^−5^
ko04075	Plant hormone signal transduction	9	13	9.34 × 10^−4^
ko00790	Folate biosynthesis	3	3	1.54 × 10^−2^
ko01040	Biosynthesis of unsaturated fatty acids	6	10	1.92 × 10^−2^
ko00591	Linoleic acid metabolism	5	8	2.67 × 10^−2^

Diffs: the number of differentially expressed proteins in metabolic pathways; Refs: the number of proteins identified in metabolic pathways.

## Supplementary Materials

Supplementary materials can be found at www.mdpi.com/1422-0067/18/12/2722/s1.
