# Supplementary material for: Proteomic Analysis Reveals Coordinated Regulation of Anthocyanin Biosynthesis through Signal Transduction and Sugar Metabolism in Black Rice Leaf
Source: Int J Mol Sci. 2017 Dec 15;18(12):2722. doi: 10.3390/ijms18122722 (PMC5751323; doi:10.3390/ijms18122722)
Supplement: Supplementary file 1 [file ijms-18-02722-s001.zip › ijms-242227 revise supplementary/ijms-242227 supplementary.docx]

**Table S1**. Differentially expressed proteins in black rice leaves at five developmental stages. (XLSX)

**Table S2.** Design of primers used for quantitative PCR analysis.

| GI Number | Gene symbol | Forward primer (5’-3’) | Reverse primer (5’-3’) |
| --- | --- | --- | --- |
| 1002244193 | HDAC | GAGAGGAAAGGAGCCGAAA | CAGGGTACACGAAAGCAAA |
| 108864047 | FBAP | GCCACCCCTGAGCAAGTATCT | CCTCCGACTGCCCACCCGACA |
| 1002306184 | JIP60 | GACGATTAGTGGAGTTGTTGGC | TGTCCTTGGTGGTGATTGTAGA |
| 1002313685 | β-gal | CTCATCTTCGTGCTACTACCCA | CTCATCTTCGTGCTACTACCCA |
| 1002282336 | CRK10 | GTGTTCTACGACCCCTGCT | CGTTCACCCACGAGATTAT |
| 1002254059 | PhyB | CTCCTCTAAACCCTTCTACG | TCAGCAATCATCCGCACA |
| 1002314010 | RFS2 | TTTCTGATCCGTTCTTGTTT | ACCTCCCACTGCCAAACTG |
| 122206953 | CFM3 | CAACCGCCTGAAACAATAA | CAGAAACTAAAATACCCCCA |


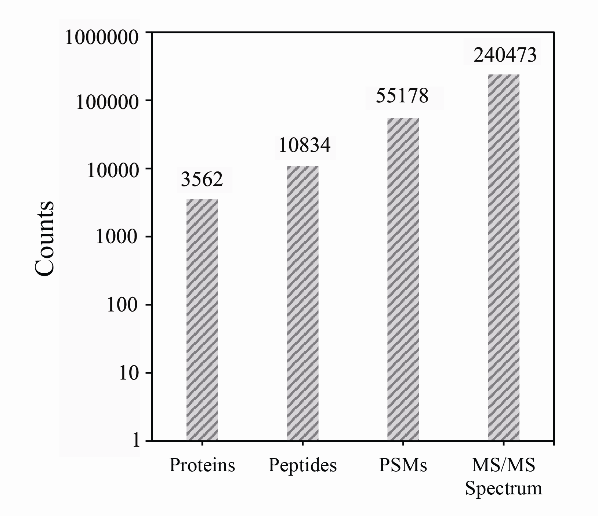


**Figure S1. The basic information of the iTRAQ experiment with black rice leaves.** An iTRAQ proteomics method was applied for comparative analysis of total proteins in leaves of black rice on 3 DAF, 7 DAF, 10 DAF, 15 DAF and 20 DAF. A total of 240,473 MS/MS fragmentation information was detected, and 55,178 MS/MS PSM could be matched with available database. A total of 10,834 peptides and 3,562 proteins were identified with the MASCOT 2.2 search engine that was embedded in Proteome Discoverer and run against the rice protein database (released in May 2016 and including 144,386 sequences from NCBI). The X axis represents the different types of MS/MS fragmentation information; the Y axis represents the total number of differential MS/MS fragments, peptides and proteins. PSM, protein spectrum matched.


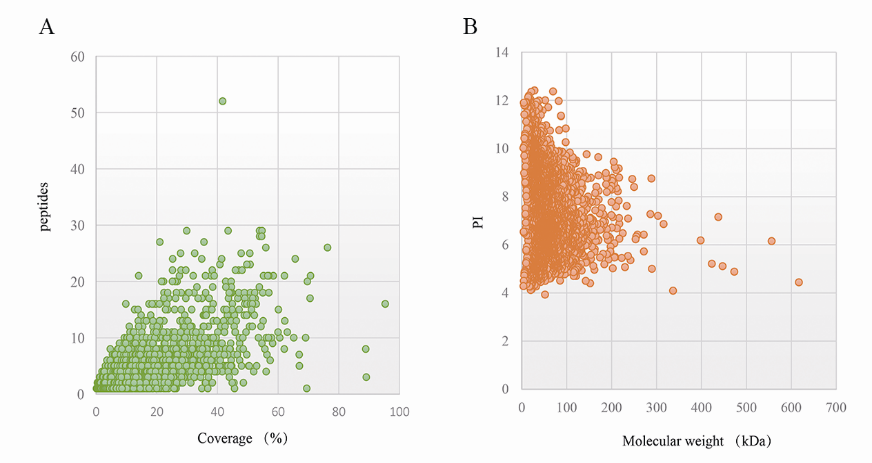


**Figure S2. Identification of protein physical and chemical characteristics.** A. Most proteins coverage rates was no more than 50% in 3,405 quantitative proteins and the number of peptides corresponding to each identified protein was less than 20%; The X axis represents the proteins coverage rates; The Y axis represents the number of peptides; B. The isoelectric point and molecular weight of most identified proteins belong to alkaline proteins. In addition, most of proteins with molecular weight greater than 100 kDa were identified. The X axis represents the molecular weight of identified proteins; the Y axis represents the the isoelectric point of each protein. PI, Isoelectric point.
